# Mated *Drosophila melanogaster* females consume more amino acids during the dark phase

**DOI:** 10.1371/journal.pone.0172886

**Published:** 2017-02-27

**Authors:** Shun Uchizono, Yumi Tabuki, Natsumi Kawaguchi, Teiichi Tanimura, Taichi Q. Itoh

**Affiliations:** 1 Graduate School of Systems Life Sciences, Kyushu University, Motooka, Fukuoka, Japan; 2 Research Fellow of Japan Society for the Promotion of Science, Tokyo, Japan; 3 Tobata High School, Tobata, Kitakyushu, Japan; 4 Department of Biology, Faculty of Science, Kyushu University, Motooka, Fukuoka, Japan; National Cancer Institute, UNITED STATES

## Abstract

To maintain homeostasis, animals must ingest appropriate quantities, determined by their internal nutritional state, of suitable nutrients. In the fruit fly *Drosophila melanogaster*, an amino acid deficit induces a specific appetite for amino acids and thus results in their increased consumption. Although multiple processes of physiology, metabolism, and behavior are under circadian control in many organisms, it is unclear whether the circadian clock also modulates such motivated behavior driven by an internal need. Differences in levels of amino acid consumption by flies between the light and dark phases of the day:night cycle were examined using a capillary feeder assay following amino acid deprivation. Female flies exhibited increased consumption of amino acids during the dark phase compared with the light phase. Investigation of mutants lacking a functional *period* gene (*per*^0^), a well-characterized clock gene in *Drosophila*, found no difference between the light and dark phases in amino acid consumption by *per*^0^ flies. Furthermore, increased consumption of amino acids during the dark phase was observed in mated but not in virgin females, which strongly suggested that mating is involved in the rhythmic modulation of amino acid intake. Egg production, which is induced by mating, did not affect the rhythmic change in amino acid consumption, although egg-laying behavior showed a *per*^0^-dependent change in rhythm. Elevated consumption of amino acids during the dark phase was partly induced by the action of a seminal protein, sex peptide (SP), on the sex peptide receptor (SPR) in females. Moreover, we showed that the increased consumption of amino acids during the dark phase is induced in mated females independently of their internal level of amino acids. These results suggest that a post-mating SP/SPR signal elevates amino acid consumption during the dark phase *via* the circadian clock.

## Introduction

In a wide range of organisms, many biological events in physiology, behavior, and metabolism are restricted to a particular time of day. These rhythmic oscillations are gated not only by environmental cues but also by the internal circadian clock [1]. The circadian clock is composed of the transcriptional-translational feedback loop (TTFL), which has been identified as the core mechanism inducing specific circadian behaviors [1,2]. In *Drosophila melanogaster*, CLOCK (CLK)/CYCLE (CYC) heterodimers (CLK/CYC) bind to E-box sequences and induce transcription of several key transcription factors, including *period* (*per*), which represses the activation of CLK/CYC to ensure circadian oscillation of the TTFL. It is well known that flies carrying a mutated version of *period* (*per*^0^) show arrhythmicity in many aspects of behavior and physiology regulated by the circadian clock [1].

Feeding behavior is one of the activities regulated by the circadian clock. Mutations in circadian genes alter the feeding rhythm in both flies and mice [3]. In *Drosophila*, for example, *takeout* (*to*), induced by *Pdp1ε*, which is one of the feedback loop components, modulates feeding behavior by conveying temporal information about the internal nutritional state [4,5]; moreover, the fat body, an important tissue for energy storage that is known to express clock genes, might also control the feeding behavior of flies [6]. Although feeding behavior is controlled by the circadian clock, the detailed mechanism through which the clock induces feeding at a particular time of day has not yet been elucidated.

Animals need to ingest appropriate nutrients depending on their internal state to maintain nutritional homeostasis, and thus their feeding behavior is dependent on their developmental, reproductive, or internal physiological state [7–10]. Animals increase their feeding preference for a particular nutrient when they are deficient in that nutrient [11–13]. Amino acids are important nutrients for development and reproduction, and a specific hunger for proteins or amino acids has been reported in several species [14–16]. In *Drosophila*, removal of amino acids from the food source prevents larval development, and the lack of only one essential amino acid prevents female flies from laying eggs [17]. It was reported recently that *Drosophila* deprived of amino acids show an enhanced preference for them [18].

In many insects, including *Drosophila*, mating causes dramatic changes in female physiology and/or behavior [19]. Female flies alter their feeding behavior towards yeast-containing food [20,21] as well as salt [10] after mating. The post-mating switch in female behavior is triggered by sex peptide (SP), a seminal protein transferred to the female during copulation [22–24]. SP activates a specific receptor, the sex peptide receptor (SPR), which is broadly expressed in the female reproductive tract and nervous system [25]. Though the period of locomotor activity rhythm does not differ between males, virgin females, and mated females, the sleep status during the light phase is dramatically changed in mated females compared with virgins, and this change results from the involvement of the post-mating SP/SPR signal [26,27]. Although feeding behavior and metabolic status are both circadian-regulated, we do not yet understand the relationship between a specific hunger for a particular nutrient and the circadian rhythm. We focused in this study on the feeding behavior after amino acid deprivation and asked whether flies consumed more amino acids at a certain time of the day, despite urgently requiring amino acids. Only mated females showed a higher level of feeding on amino acids in the dark phase than in the light phase; wild-type (CS) males, virgin CS females, and *per*^0^ flies did not. This suggested that only mated females had a specific reason for changing their amino acid consumption over a day. We then investigated whether the post-mating responses (PMRs) of females were related to the time-dependent consumption of amino acids. Time dependency in amino acid consumption was still observed in egg production mutants, indicating that egg-laying behavior itself is not important in increasing amino acid consumption during the dark phase of the 12 h light: 12 h dark (LD) cycle. The flies lacking the SP/SPR signal showed partly diminished amino acid consumption during the dark phase, compared with CS mated females. This suggests that the post-mating SP/SPR signal promotes amino acid consumption during the dark phase by interacting with the circadian clock.

## Materials and methods

### Fly stocks

Canton-Special (CS) was used as a wild-type control strain in all experiments. *per*^0^ [28] was used as a representative clock gene mutant strain. *SP*^0^/Δ^130^ flies were generated by crossing *SP*^0^/*TM3*,*Sb* to Δ^130^/*TM3*,*Sb* stocks, and *SP* null mutant males [29] were used in experiments. A *SPR* mutant strain, *Df(1)Exel6234*, was donated by Y. J. Kim (Gwangju Institute of Science and Technology, Gwangju, Korea). *ovo*^*D1*^/CS sterile females were generated by crossing *ovo*^*D1*^ males to CS females; the *ovo*^*D1*^ strain is maintained with a compound-X chromosome and was obtained from the Bloomington *Drosophila* Stock Center. All fly stocks were raised on a standard cornmeal-agar-yeast-glucose medium (SM) under LD cycles at 25 °C.

### Amino acid deprivation

Adult flies were collected within 24 h of eclosion and raised on an amino acid-deficient glucose [aa(-)] medium containing 90.08 g glucose, 1 g sodium hydrogen carbonate, 0.7 g potassium dihydrogen phosphate, 3.9 g di-potassium hydrogen phosphate, 0.2 g magnesium sulfate, 0.1 g phosphatidylcholine (dissolved in 1 ml ethanol), 2 ml propionic acid, and 9 g agar in 1L water (modified from [30]). Female and male flies were raised together on the medium, and flies were transferred to fresh aa(-) medium every other day.

### Chemicals

Chemicals used in the aa(-) medium were obtained from the following sources: D-glucose was obtained from Sigma-Aldrich (St. Louis, USA); sodium hydrogen carbonate and magnesium sulfate were obtained from Wako Pure Chemical Industries (Osaka, Japan); potassium dihydrogen phosphate, di-potassium hydrogen phosphate, L-α-phosphatidylcholine, propionic acid, and agar (purified powder) were all obtained from Nacalai Tesque (Kyoto, Japan).

The stock of sodium-free amino acid mixture, based on a previous study [30] and used in all experiments, was made up as follows: 0.5 mM L-tyrosine (35709, Nacalai), 2 mM L-(+)-arginine monohydrochloride (03323, Nacalai), 3.5 mM L-aspartic acid (03503, Nacalai), 4 mM L-glutamic acid (16911, Nacalai), 5 mM L-tryptophan (35607, Nacalai), and 10 mM each of L-α-alanine (01115, Nacalai), L-asparagine monohydrate (03427, Nacalai), L-cysteine hydrochloride monohydrate (039–05274, Wako), L-glutamine (16919, Nacalai), glycine (17109, Nacalai), L-histidine monohydrochloride monohydrate (18119, Nacalai), L-isoleucine (I2752, Sigma), L-leucine (20327, Nacalai), L-lysine monohydrochloride (20809, Nacalai), L-methionine (21719, Nacalai), L(-)-phenylalanine (161–01302, Wako), L(-)-proline (161–04602, Wako), L-serine (195–00404, Wako), L(-)-threonine (204–01322, Wako), and L-valine (228–00082, Wako). The stock amino acid mixture was used at 1/10 or 1/5 dilution. Food dyes (Food Blue No. 1 and Food Red No. 106) were obtained from Tokyo Chemical Industry Co. (Tokyo, Japan).

### CAFE assay

The capillary feeder (CAFE) assay was modified from the previously reported method [31,32] as follows: four microcapillary tubes were inserted in a Buzz Plug, which was placed into an experimental vial with a piece of wet Kimwipe on the bottom. In a two-choice assay, two of the capillary tubes were filled with 10 mM or 50 mM glucose solution colored with 250 mg/L red food dye, and the other two tubes were filled with 1/10 amino acid mixture or 1/5 amino acid mixture and 50 mM glucose colored with 125 mg/L blue food dye. In a no-choice assay, all capillaries were filled with either glucose solution or the amino acid mixture.

Male and female flies, which eclosed within a 24 h period, were kept together on aa(-) medium for 2 or 3 days or on SM for 3 days under LD cycles. Separate groups of 10 male or 10 female flies were then transferred to fresh aa(-) medium or SM and kept for another 2 or 1 day(s). Virgin females eclosing within a 12 h period were kept alone on aa(-) medium or SM for 4 days. Four days after eclosion, flies without anesthesia were introduced into experimental vials, and the intake of glucose and amino acids during the light and dark phases was measured. Three control vials without flies were included in each experiment to measure the amount of evaporation; the mean amount of evaporation was subtracted from the decrease in each tested tube. In the two-choice assay for flies without amino acid deprivation, amino acid intake was normalized by subtracting the intake of 50 mM glucose from the intake of 1/5 amino acid mixture and 50 mM glucose in each vial.

### Two-choice preference test

The two-choice preference test was performed as previously described [18]. A piece of chromatography paper was soaked with 150 μl 10 mM glucose solution colored with 250 mg/L red food dye, and another piece of chromatography paper was soaked with 150 μl amino acid mixture colored with 125 mg/L blue food dye. The food dyes do not influence taste preference at the concentration used in this assay [18,33]. Approximately 50–60 flies were raised on aa(-) medium for 2 days, and then tests were performed at four Zeitgeber times (ZTs) under LD cycles or at four circadian times (CTs) in constant darkness (DD) or constant light (LL). The flies were placed in a Petri dish containing chromatography papers bearing glucose and amino acids and tested for 2 h. After the test period, flies were frozen and their abdominal colorings were observed under a compound stereomicroscope. Flies were classified into red (R), blue (B), purple (M), and uncolored (O) groups, and feeding ratios (FRs) of glucose and amino acids were calculated using the following formulae:

FR of glucose: (*N*^R^ +*N*^M^/2) / (*N*^R^ + *N*^B^ + *N*^M^ + *N*^O^)
FR of amino acids: (*N*^B^ +*N*^M^/2) / (*N*^R^ + *N*^B^ + *N*^M^ + *N*^O^)

where *N*^R^, *N*^B^, *N*^M^, and *N*^O^ were the number of flies with red, blue, purple, and uncolored abdomens, respectively.

### Measurement of circadian rhythms and sleep

Flies were kept on SM under LD cycles at 25°C for 2–5 days post-eclosion. Virgin and mated females were then loaded individually into 5×65 mm glass capillary tubes containing agar gel with 100 mg/ml glucose [34]. A *Drosophila* Activity Monitor system (Trikinetics; Waltham, MA, US) was used to record locomotor activity for 4 days under LD cycles, followed by 7 days in DD. The periods were calculated by chi-square periodogram analysis with the significance level set to α = 0.05 [35], programmed by the Matlab R2007b software (MathWorks Inc.). Flies with a chi-square statistic ≥ 10 over the significance line were scored as rhythmic [36]. The 4 days of data recorded under LD cycles were used to assess waking, sleep, and walking activity. The data were analyzed using a custom-written Excel macro [37]. Sleep was defined as ≥ 5 min of inactivity (zero infrared beam crossings). Walking activity was defined as the number of times the infrared beam was crossed.

### Oviposition assay

Female flies eclosing within 24 h were collected and placed, together with male flies, on SM or aa(-) medium for 4 days under LD cycles at 25 °C. Groups of 3–5 mated females, together with 1–2 males, which had been kept on SM or aa(-) medium, were then placed in a Petri dish (55 mm diameter) containing SM or aa(-) medium. Dyes (62.5 mg/L blue food dye and 125 mg/L red food dye) were mixed with SM to enable easy recognition of eggs on the medium. The number of eggs laid during light and dark phases was counted under a compound stereomicroscope. To calculate the number of eggs laid by individual females, the total number of eggs on the plate was divided by the number of surviving females at the end of the assay.

## Results

### Females ingested more amino acids in the dark phase than in the light phase

**Fig 1:**
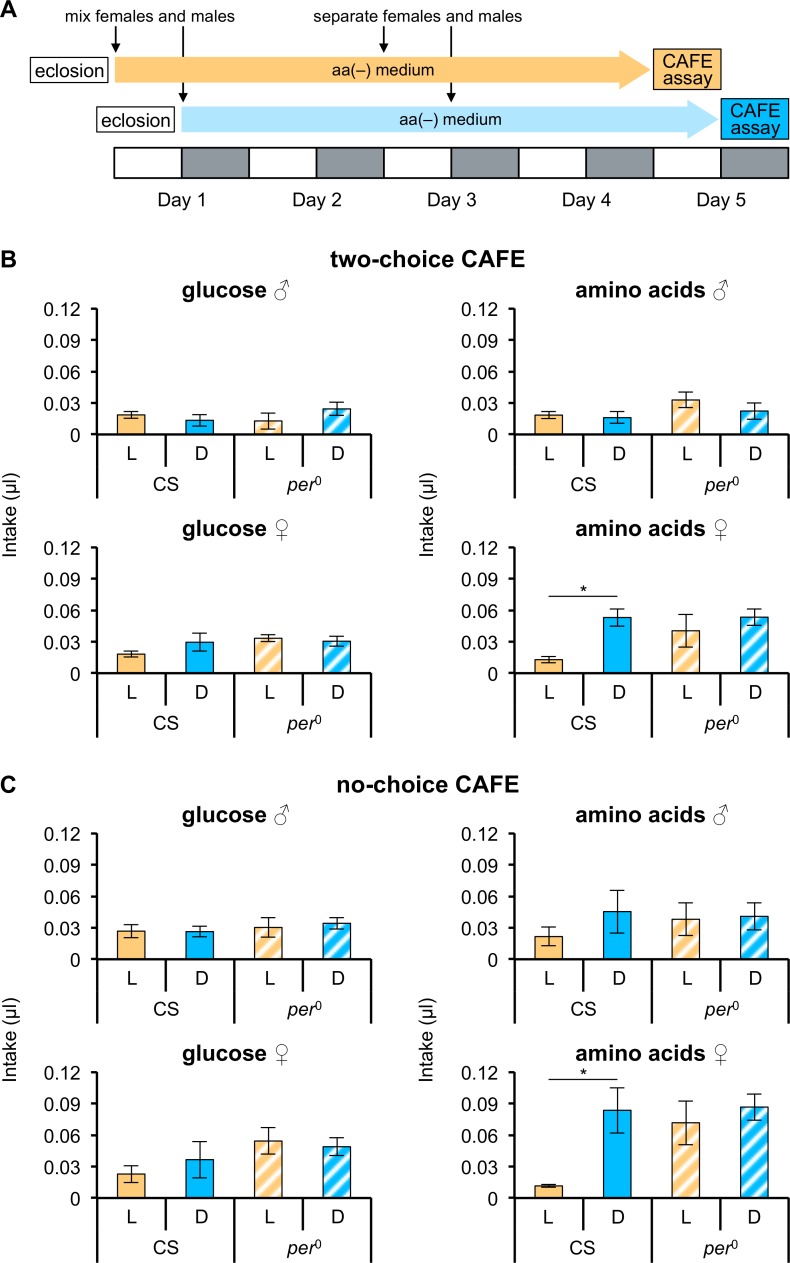
Females ingest an increased quantity of amino acids during the dark phase. The experimental scheme is indicated in (A). Each 12 h light (L) phase is shown by a white box and each 12 h dark (D) phase by a gray box. Intake of glucose and amino acids during L and D phases was quantified using the capillary feeder (CAFE) assay. Assays were performed in the two-choice situation (B) between glucose and amino acids (n = 3 or 4 trials) and in the no-choice situation (C) (n = 3 or 4 trials). The quantity of intake per single fly is shown. Intake during L and D phases is indicated by orange and blue bars, respectively; filled bars represent CS flies; hatched bars represent *per*^0^ flies. Error bars indicate SEM. *p < 0.05 for comparisons between L and D phases using the Student’s *t*-test.

Deprivation of amino acids induces a specific appetite, and thus flies increase amino acid consumption to meet their internal needs [18]. The two-choice CAFE assay (between glucose and amino acids) was used to quantify consumption of glucose and amino acids, and thus determine whether amino acid-deprived flies ingested more amino acids at a constant rate across the day. CS flies were kept on aa(-) medium under LD cycles for 4 days before the assays (Fig 1A).

No difference across a day was observed in the consumption of either glucose or amino acids in CS males (Fig 1B); CS females, however, significantly increased their amino acid intake during the dark phase, relative to the light phase, although their glucose intake was not significantly different between the light and dark phases of the LD cycle (Fig 1B). This suggests that females alter their intake of amino acids between the light and dark phases.

We wondered whether clock genes were involved in the differences in consumption of amino acids between the light and dark phases, and thus measured glucose and amino acid intakes in a strain carrying a mutant clock gene (*per*^0^). Glucose and amino acids intakes in *per*^0^ flies were constant between the light and dark phases in both sexes, suggesting that consumption of amino acids in females is under control of the circadian clock.

Since the assay gave flies a choice between glucose and amino acids, we wondered whether the presence of glucose was affecting the level of amino acid intake. We therefore performed a no-choice CAFE assay of glucose and amino acid consumption. The glucose intake of CS flies was unchanged between the light and dark phases, but the amino acid intake of females increased during the dark phase relative to the light phase (Fig 1C), which is the same result as that obtained in the two-choice CAFE assay. Likewise, there were no significant differences between the light and dark phases in glucose and amino acid intakes in *per*^0^ flies. We concluded, therefore, that glucose intake was independent of the change between the light and dark phases in amino acid intake. Notably, the total amount of feeding throughout the day was greater in *per*^0^ than in CS, which is consistent with a recent report [38]. Taken together, these results show that female flies ingested greater quantities of amino acids during the dark phase, and that amino acid consumption was controlled by the circadian clock.

### Feeding preference for glucose and amino acids does not show a circadian pattern

**Fig 2:**
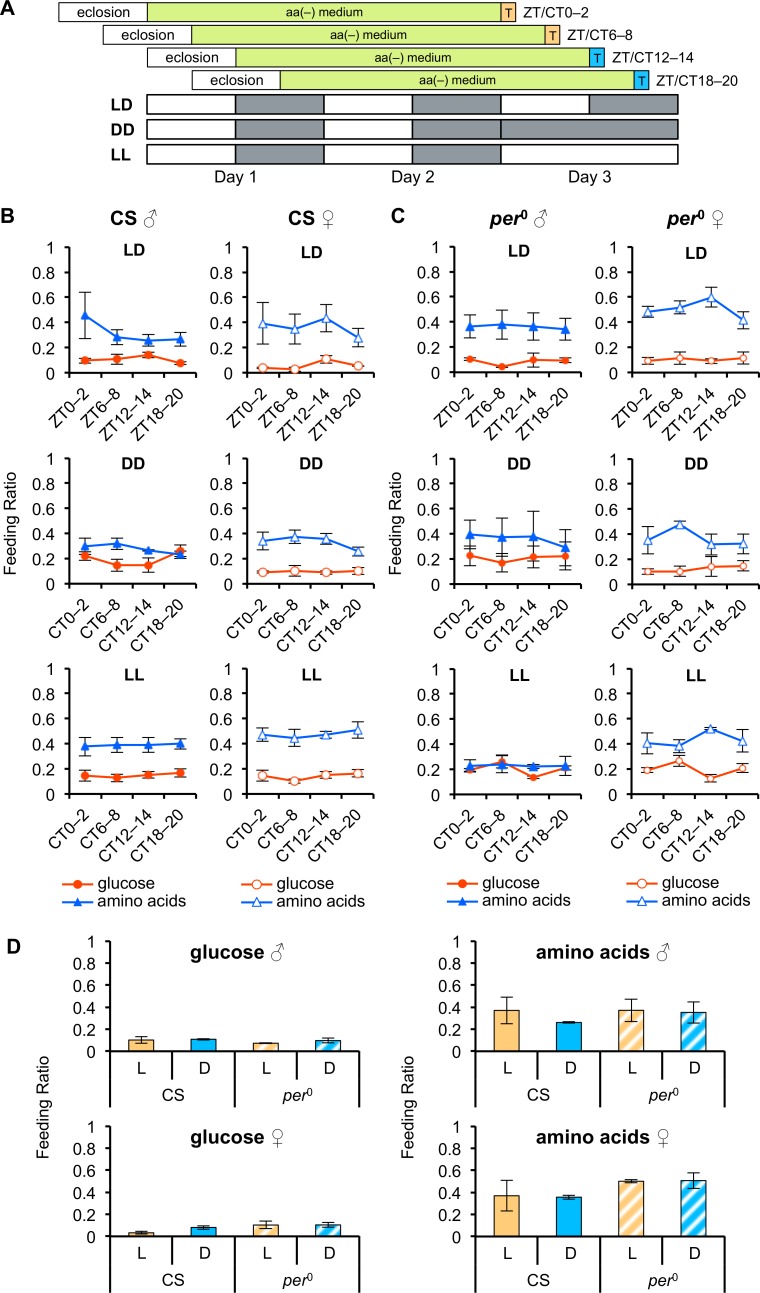
Taste preference for amino acids does not show daily rhythmicity. (A) Diagram of two-choice preference tests. The flies were kept on aa(-) medium for 2 days, and then offered a choice between glucose or amino acid mixture for 2 h (indicated as T) at each time point under LD cycles, DD, or LL (n = 3 or 4 in quintuple). Each light (L) phase is shown by a white box and each dark (D) phase by a gray box. (B and C) Feeding ratios (FRs) between glucose (red lines) and amino acids (blue lines) of CS (B) and *per*^0^ (C) flies are shown at four Zeitgeber (ZT) or circadian (CT) time points. *p* > 0.05 for all comparisons between feeding ratios across the four time points under LD, DD, and LL using one-way ANOVA. (D) Feeding ratios at ZT 0–2 and 6–8 in (B) and (C) were integrated as the feeding ratio in the L phase (orange bars). Feeding ratios at ZT 12–14 and 18–20 in (B) and (C) were integrated as a feeding ratio in the D phase (blue bars); filled bars represent CS flies; hatched bars represent *per*^0^ flies. *p* > 0.05 for all comparisons made between L and D phases using Student’s *t*-test. Error bars indicate SEM.

Several previous studies have demonstrated that deprivation of yeast or amino acids increases the feeding preference for yeast or amino acids, as well as increased consumption [18,20]. To determine whether the feeding preference for amino acids also changed across a day in females deprived of amino acids, we examined the feeding preference for amino acids at four different time points (ZT 0–2, 6–8, 12–14, and 18–20) across a 24 h day using the two-choice preference test (Fig 2A). Both male and female CS flies kept on aa(-) medium under LD cycles preferred amino acids to glucose (Fig 2B and 2D), as previously reported [18]. Surprisingly, unlike the level of intake, the FR of amino acids (percentage of flies that preferred amino acids to glucose), as well as that of glucose, in both male and female CS flies remained unchanged across the four different time points (Fig 2B; one-way ANOVA, *p* > 0.05).

To test if there was a masking effect of light on the feeding preference during the light phase, we repeated the two-choice tests in DD at four CTs (0–2, 6–8, 12–14, and 18–20). Again, neither males nor females showed any significant differences in the FRs of glucose and amino acids across the four CTs in DD (Fig 2B; one-way ANOVA, *p* > 0.05), suggesting that there was no masking effect of light on the feeding preference. In addition, to determine whether there was a masking effect of dark on the feeding preference during the dark phase, the two-choice tests were repeated in constant light (LL). In LL, flies of both sexes showed constant FRs of glucose and amino acids at the four CTs (Fig 2B; one-way ANOVA, *p* > 0.05), demonstrating no masking effect of dark on the feeding preference for glucose and amino acids.

To explore whether clock genes were involved in keeping the feeding preference constant across a day, we performed pairwise tests with *per*^0^ flies (Fig 2C and 2D). As with CS flies, the FRs of glucose and amino acids remained constant in *per*^0^ flies across four different time points in LD, DD, and LL (Fig 2C; one-way ANOVA, *p* > 0.05). For comparisons between levels of intake (Fig 1) and FRs, we integrated the FRs at ZT 0–2 and 6–8 as the light (L) phase, and at ZT 12–14 and 18–20 as the dark (D) phase (Fig 2D). No significant differences were observed between light and dark phases in either CS or *per*^0^ flies. The feeding preferences for glucose and amino acids thus appeared to be independent of light and dark signals, and even of the circadian clock.

### Mating induces an increased consumption of amino acids during the dark phase

As only CS females showed a difference between the light and dark phases in the level of amino acid intake, it is possible that a specific behavior controlled by the circadian clock underlies amino acid intake in these flies. We first focused on levels of sleep and locomotor activity in CS and *per*^0^ females. In both CS and *per*^0^ females, the total amount of time spent awake was higher in the light phase than in the dark phase, while the total amount of sleep in the light phase was lower than in the dark phase (S1A and S1B Fig). CS females had significantly higher activity levels during the dark phase than the light phase; by contrast, the activity levels of *per*^0^ flies were significantly higher during the light phase than the dark phase (S1C Fig). Although we cannot rule out the possibility that higher activity in the light phase induced increased consumption of amino acids during the light phase in *per*^0^ females, locomotor activity is unlikely to be the main factor inducing the rhythmic consumption of amino acids because *per*^0^ females showed no significant difference in amino acid intake between the light and dark phases. These results imply the levels of sleep and locomotor activity might not be the specific behavior underlying the rhythmic intake of amino acids.

**Fig 3:**
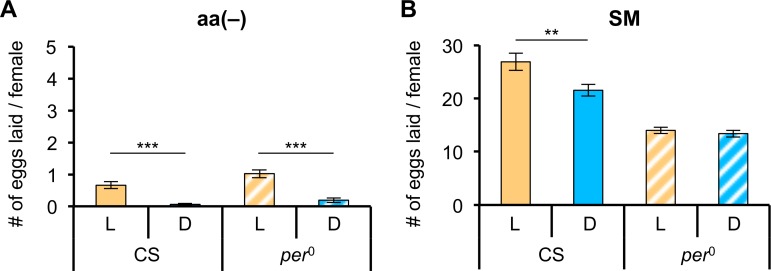
Number of eggs laid in the light and dark phases differs for CS flies. The oviposition assay was performed for 12 h starting at either ZT 0 (for the light phase) or ZT 12 (for the dark phase). L and D represent results obtained during the light (orange bars) and dark (blue bars) phases, respectively; filled bars represent CS flies; hatched bars represent *per*^0^ flies. (A) The mean number of eggs laid during L and D phases on aa(-) medium by an amino acid-deprived female (n = 20). (B) The mean number of eggs laid during L and D phases on a standard cornmeal-agar-yeast-glucose medium (SM) by a female raised on SM (n = 20). Error bars indicate SEM. **p < 0.01 and ***p < 0.001 for comparisons between L and D phases using the Student’s *t*-test.

Next, we focused on egg-laying behavior of CS and *per*^0^ females. We counted the numbers of eggs laid by females kept on aa(-) medium for 4 days (Fig 3A; see materials and methods). As the mean number of eggs laid per female under these conditions was less than one, consistent with an earlier report [17], we repeated the assay using CS and *per*^0^ females kept on SM for 4 days (Fig 3B). The mean number of eggs laid per female in the test using SM was greater than 10. The number of eggs laid by CS females in the light phase was significantly higher than in the dark phase, whereas there was no difference in the mean number of eggs laid by *per*^0^ females in the light and dark phases. These results imply that the difference in amino acid intake between light and dark phases might be related to egg-laying behavior.

**Fig 4:**
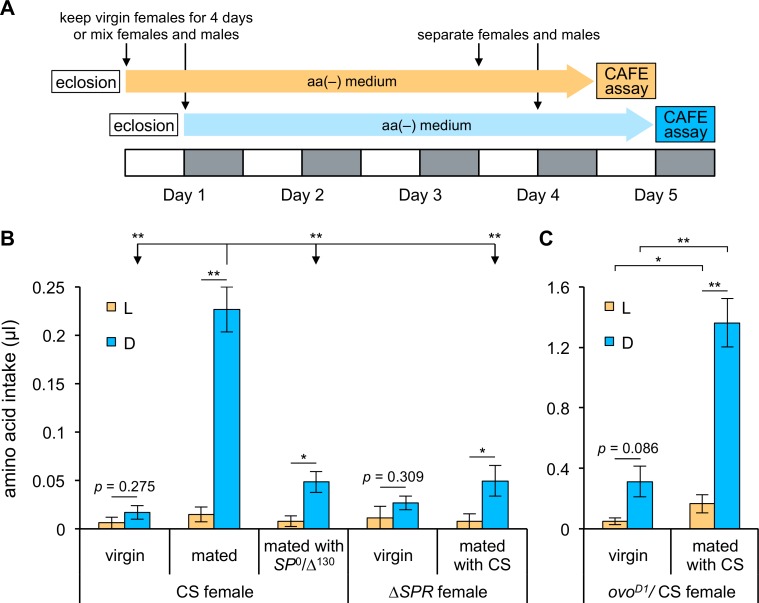
A post-mating signal elevates amino acid consumption during the dark phase. (A) The experimental scheme for the CAFE assays. Each L phase is shown by a white box and each D phase by a gray box. (B and C) Amino acid consumption during L (orange bars) and D (blue bars) phases was quantified using no-choice CAFE assays with the following strains: virgin CS females and CS females mated with CS or *SP*^0^/Δ^130^ males (B; n = 3 or 4 trials); virgin *Df(1)Exel6234* (shown as Δ*SPR*) females and *Df(1)Exel6234* females mated with CS males (B; n = 4 trials); and virgin *ovo*^*D1*^/CS females and *ovo*^*D1*^/CS females mated with CS males (C; n = 4 trials). Intake per single fly is shown. Error bars indicate SEM. *p < 0.05 and **p < 0.01 for comparisons between L and D phases for each type of female in (B) and (C) using the Student’s *t*-test. *p* > 0.05 for all comparisons during L phase among females in (B) using one-way ANOVA. *p < 0.05 and **p < 0.01 for all comparisons during D phase among females in (B) using one-way ANOVA followed by *post hoc* Bonferroni/Dunn test. *p < 0.05 and **p < 0.01 for comparisons between *ovo*^*D1*^/CS virgin and mated females in (C) using the Student’s *t*-test.

As mating elicits increased egg laying [29] and amino acid consumption is important for egg laying by mated females (Fig 3B), we hypothesized that mating facilitated amino acid consumption during the dark phase by interacting with the clock that caused eggs to be laid in a circadian manner. We first examined whether virgin CS females showed differences in amino acid consumption between the light and dark phases of the LD cycle using the no-choice CAFE assay. To examine the effect of mating more precisely, mated CS females were also tested again (Fig 4A) by placing females with males for 1 day longer than in the previous no-choice CAFE assay (Fig 1A). As we expected, the consumption of amino acids by virgin CS females during the dark phase was dramatically lower than that of mated CS females; moreover, virgin CS females did not show differences in amino acid consumption between the light and dark phases (*t*-test, *p* = 0.275).

To determine whether the post-mating switch regulating amino acid consumption involved the SP and SPR pathway, amino acid consumption by CS females mated with *SP* mutant males and by *SPR* mutant females mated with CS males was measured using the no-choice CAFE assay (Fig 4B). Both groups of females still showed significant differences in amino acid consumption between the light and dark phases, although *p* values were greater than 0.01 (CS females mated with *SP* mutant males: *t*-test, *p =* 0.015; Δ*SPR* females mated with CS males: *t*-test, *p =* 0.042). The levels of amino acid intake of CS females mated with SP mutant males and mated *SPR* mutant females were significantly lower than that of mated CS females only in the dark phase. In addition, virgin *SPR* mutant females did not show differences in amino acid consumption between the light and dark phases (*p* = 0.309 with *t*-test), as previously observed in CS virgin females. These results indicated that mating must be the trigger to increase amino acid consumption during the dark phase of the LD cycle.

To determine whether the increase in amino acid consumption by mated females during the dark phase was due to amino acid deprivation resulting from egg laying, we examined consumption of amino acids by females carrying the dominant *ovo*^*D1*^ mutation (Fig 4C); such females lack the ability to produce eggs due to an arrest in egg development. Significant differences in amino acid consumption between the light and dark phases were still observed in mated *ovo*^*D1*^ females, although the level of intake of *ovo*^*D1*^ females was much greater than that of mated CS females. Elevated food consumption by *ovo*^*D1*^ females has been previously reported to result from an increase in the volume of food consumed per proboscis extension [39]. We also tested virgin *ovo*^*D1*^ females and found no significant differences in intake between the light and dark phases (*t*-test, *p* = 0.086), similar to the result obtained from virgin CS females. These results suggest that egg-laying behavior is not itself necessary to produce the difference in amino acid consumption between the light and dark phases.

To rule out the possibility that the absence of differences in amino acid consumption by virgin CS and *ovo*^*D1*^ females between the light and dark phases resulted from behavioral arrhythmicity (as in *per*^0^ females), we examined their locomotor activity rhythms (Table 1). While all the *per*^0^ females tested showed arrhythmic locomotor activity, the rates of rhythmicity in CS and *ovo*^*D1*^ virgin females were greater than 90% and the mean period of the locomotor activity rhythm was within 0.5 h of 24 h, which is considered the wild-type locomotor phenotype of *Drosophila*. The locomotor activity rhythms of *SP* and *SPR* mutants also had normal periods (Table 1). These results strongly indicate that an increase in amino acid consumption during the dark phase is triggered by mating, and that signaling through the SP/SPR pathway promotes amino acid consumption during the dark phase by modulation of the circadian clock.

**Table 1:** Free-running periods of the flies used in the CAFE assay.

Lines	Period	SEM	N	R%
*per*^0^ female (mated with *per*^0^ male)	-	-	27	0
CS virgin female	24.23	0.07	32	93.75
CS female (mated with CS male)	23.87	0.06	30	86.67
CS female (mated with *SP*^0^/Δ^130^ male)	24.22	0.05	33	96.97
*Df(1)Exel6234* (Δ*SPR*) virgin female	24.06	0.05	31	83.87
*Df(1)Exel6234* (Δ*SPR*) female (mated with CS male)	24.12	0.07	33	100
*ovo*^*D1*^/CS virgin female	24.06	0.04	34	97.06
*ovo*^*D1*^/CS female (mated with CS male)	24.47	0.07	33	96.07

N indicates number of flies analyzed. R% indicates percent flies with detectable rhythmicity.

**Fig 5:**
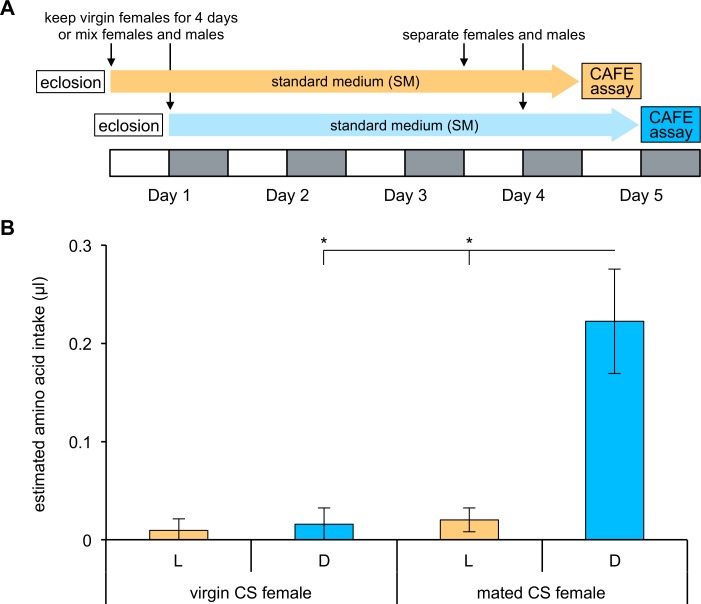
Mated females increase amino acid consumption during the dark phase without amino acid deprivation. (A) The experimental scheme for the CAFE assays. Flies were tested without deprivation of amino acids. Each L phase is shown by a white box and each D phase by a gray box. (B) Amino acid consumption during L (orange bars) and D (blue bars) phases was quantified using two-choice CAFE assays between 50 mM glucose and 50 mM glucose containing 1/5 amino acid mixture in virgin and mated CS females (n = 6 trials). Intake of 50 mM glucose alone was subtracted from intake of 50 mM glucose containing 1/5 amino acid mixture, and the value was subsequently divided by the number of flies in each vial. The mean value is shown as “estimated amino acid intake”. Error bars indicate SEM. *p < 0.05 for comparisons between L and D phases and between virgin and mated females using the Student’s *t*-test.

Finally, we asked whether the increase of amino acid consumption during the dark phase is induced after mating regardless of the deprivation. To this end, we examined the amino acid intake of females without deprivation of amino acids by the two-choice CAFE assay (Fig 5A; see materials and methods). Mated females without amino acid deprivation consumed more amino acids during the dark phase than the light phase in accordance with the case of mated females deprived of amino acids (Fig 5B; *t*-test, *p =* 0.010). Moreover, the elevated amino acid consumption during the dark phase was not observed in virgin females without the deprivation, and their amino acid intake during the dark phase was significantly lower than that of mated females (*t*-test, *p =* 0.010). Taken together, these results suggest that mating elicits amino acid consumption during the dark phase in females without depending on their internal level of amino acids.

## Discussion

The feeding preference of *Drosophila* for proteins or amino acids increases in response to deprivation of these nutrients [18,20,21]. Given that there is a daily rhythm in a variety of metabolic, physiological, and behavioral processes, we wondered whether flies showed diurnal changes in feeding behavior for particular nutrients, such as amino acids, required for those processes. We found that mated females showed such a diurnal change, increasing consumption of amino acids during the dark phase throughout the day.

Our two-choice and no-choice CAFE assays showed that the consumption of amino acids was elevated during the dark phase only in mated females. Thus, amino acid consumption appears to be regulated in mated females during the light and dark phases. On the other hands, both male and female flies in CS and *per*^0^ did not change their feeding preference for either glucose or amino acids across a 24 h day in our two-choice preference tests. Since there was a disparity between constant feeding preference and fluctuating consumption of amino acids across a day, the circadian clock may modulate feeding behavior independently of pathways associated with appetite.

Interestingly, a previous study using the CAFE assay reported that *w*^1118^ flies showed a peak of sucrose consumption in the early daytime [6], whereas we did not observe any change in glucose consumption between the light and dark phases. We compared food consumption over 12 h for each of the light and dark phases (i.e., both phases include the dawn, which is around the peak time of sucrose consumption). Even if there were an underlying diurnal rhythm in glucose intake, it is possible that total glucose consumption might show no difference between the two phases due to the long measurement interval in our tests. Nevertheless, mated females exhibit the difference in the consumption of amino acids between the two phases, implying glucose and amino acid consumption are independently regulated by the circadian clock.

Mating drastically changes the physiological status of females and modifies feeding behavior to meet the internal demands for nutrients [10,19–21]. Food consumption is up-regulated after mating, depending on egg production [24,40]. Mating and egg-laying behavior have been shown to be rhythmic [41], and we also observed a difference between the light and dark phases in the average number of eggs laid per female over 12 h. We furthermore found that there was no difference between the light and dark phases in the average number of eggs laid by *per*^0^ females. McCabe and Birley, however, reported that *per*^0^ females still showed an egg-laying rhythm [42], which appears to contradict our results. As periodicity of the egg-laying rhythm was disrupted in LN_v_-ablated flies [43] and *per*^0^ females kept under DD showed inconsistent periods of egg laying [44], it is possible that the periodicity of the egg-laying rhythm of *per*^0^ females is altered or has a greater variance, even under LD conditions. Thus, if the peak and trough times of the egg-laying rhythm of *per*^0^ females fall at the lights-on and -off times, respectively, the average number of eggs laid per female over 12 h (our experimental condition) is likely to be equal in the light and dark phases. Alternatively, although we measured numbers of eggs laid over 24 h, these data might not include the peak if the period in *per*^0^ females is longer than 24 h. Nevertheless, our observation that *per*^0^ females showed no differences in either the level of amino acid intake or egg laying between light and dark phases implies that the link between amino acid intake and egg-laying behavior is *via* the clock gene, *per*.

Given the correlated rhythmicity of amino acid intake and egg laying, there are three possibilities: (i) the egg-laying rhythm controlled by the circadian clock drives the rhythmic consumption of amino acids; (ii) the circadian clock regulates the level of amino acid intake to render egg laying rhythmic; or (iii) the clock synchronizes the two behaviors in parallel. Our observation of increased amino acid consumption during the dark phase by *ovo*^*D1*^ females disproves the first hypothesis. In addition, the mean number of eggs laid by *per*^0^ females was lower than that by CS females regardless of light and dark phases. A previous report that “wrong time” feeding could reduce a fly’s reproductive capability [44] supports hypothesis (ii), namely, that an arrhythmic intake of amino acids results in the reduction in the number of eggs laid by *per*^0^ flies; however, it is not possible yet to reach a definite conclusion on whether hypothesis (ii) or (iii) is valid. Additionally, the increased intake of yeast following mating was recently reported to result from the action of SP on SPR-expressing SP sensory neurons (SPSNs) in the reproductive tract, which is also necessary for the modulation of egg laying [10]. The circadian clock may interact with the mating signal downstream of these SPSNs to induce rhythmic feeding on amino acids and egg laying. Further studies are needed to identify which clock-regulated neuron interacts with the mating signal to modulate amino acid intake and egg-laying behavior.

## Supporting information

S1 Fig Females show differences in sleep status and locomotor activity between the light and dark phases. Behaviors of CS and *per*^0^ flies were recorded under LD cycles for 4 days at 25 °C. L and D represent the results obtained during light (orange bars) and dark (blue bars) phases, respectively; filled bars represent CS flies; hatched bars represent *per*^0^ flies. (A) the total time spent awake (min) over 4 days, (B) the total amount of sleep time (min) over 4 days, (C) the total walking activity over 4 days (times) in CS (n = 32) and *per*^0^ (n = 31) females. Error bars indicate SEM. *** *p* < 0.001 for comparisons between L and D phases using the Student’s *t*-test.
(TIF)
