# Supplementary figures and images for: Mated Drosophila melanogaster females consume more amino acids during the dark phase
Source: PLoS One. 2017 Feb 27;12(2):e0172886. doi: 10.1371/journal.pone.0172886 (PMC5328406; doi:10.1371/journal.pone.0172886)

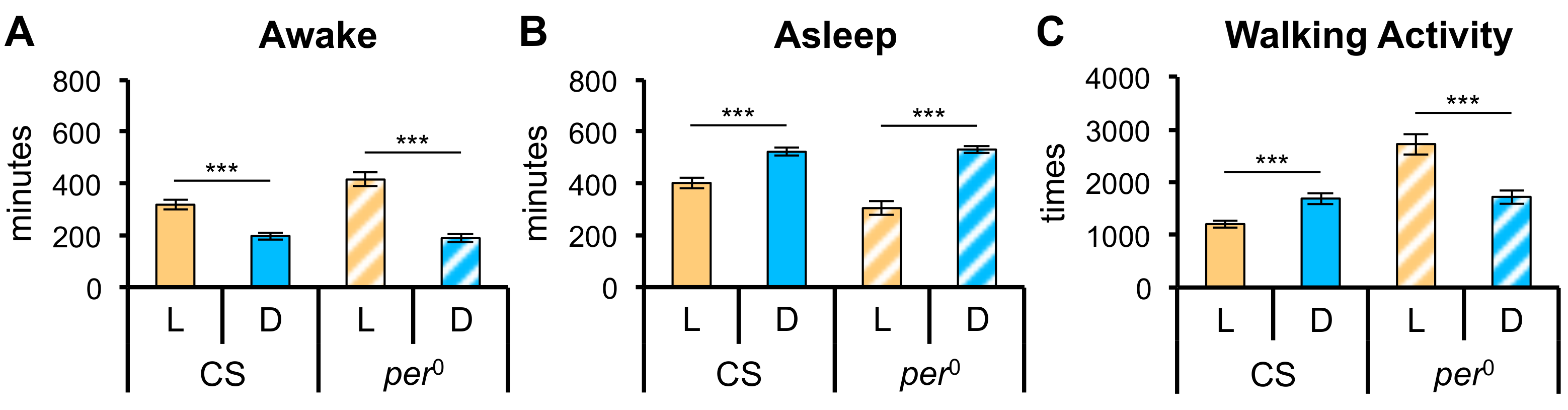

Supplement: S1 Fig — Behaviors of CS and per0 flies were recorded under LD cycles for 4 days at 25 °C. L and D represent the results obtained during light (orange bars) and dark (blue bars) phases, respectively; filled bars represent CS flies; hatched bars represent per0 flies. (A) the total time spent awake (min) over 4 days, (B) the total amount of sleep time (min) over 4 days, (C) the total walking activity over 4 days (times) in CS (n = 32) and per0 (n = 31) females. Error bars indicate SEM. *** p < 0.001 for comparisons between L and D phases using the Student’s t-test. (TIF) [file pone.0172886.s001.tif]
